# Recurrent Catatonia During Longitudinal Evaluation of Possible Seronegative Autoimmune Encephalitis: A Case Report

**DOI:** 10.7759/cureus.114401

**Published:** 2026-08-12

**Authors:** Garrison Burky

**Affiliations:** 1 Psychiatry, Summa Health, Akron, USA

**Keywords:** autoimmune encephalitis, catatonia, electroconvulsive therapy (ect), ovarian teratoma, psychosis, seronegative autoimmune encephalitis

## Abstract

Autoimmune encephalitis, primary psychiatric illness, seizure disorders, and catatonia can produce overlapping neuropsychiatric manifestations that complicate diagnosis and management, particularly when autoimmune encephalitis is suspected without detectable neuronal autoantibodies. We describe a woman in her early 20s with prior resection of a mature cystic ovarian teratoma and a reported clinical diagnosis of possible seronegative autoimmune encephalitis without documented serologic confirmation who experienced recurrent mania, psychosis, reduced oral intake, withdrawal, psychomotor slowing, and catatonic features across several hospitalizations in the context of a co-occurring seizure disorder. Her evaluations included neuroimaging, lumbar puncture, electroencephalography, evaluation for recurrent ovarian teratoma, and repeated neurologic consultation. Intravenous immunoglobulin was administered during one hospitalization because an autoimmune encephalitis relapse remained under consideration. Subsequent magnetic resonance imaging, pelvic imaging, and electroencephalography did not demonstrate objective evidence supporting active inflammatory recurrence. Catatonia became increasingly clinically actionable because symptoms repeatedly improved after lorazepam administration, prior malignant catatonia had responded to an extended course of electroconvulsive therapy, and lorazepam tapering was followed by recurrent psychiatric and catatonic deterioration with improvement after treatment was resumed. Although benzodiazepine responsiveness supported the clinical diagnosis of catatonia, it was interpreted within the broader clinical context and was not considered diagnostic in isolation. This case illustrates the value of longitudinal syndromic reassessment when etiologic certainty remains limited. Recognition and treatment of episodes clinically concerning for catatonia should not be delayed while autoimmune, seizure-related, medication-related, and primary psychiatric contributors continue to be evaluated.

## Introduction

Autoimmune encephalitis can present with psychiatric and neurologic manifestations, including psychosis, behavioral change, seizures, cognitive dysfunction, movement abnormalities, autonomic instability, and catatonia [1,2]. Psychiatric symptoms may precede more recognizable neurologic findings, creating substantial diagnostic difficulty early in the illness [1]. This uncertainty is especially pronounced when known neuronal autoantibodies are not detected. Under the Graus clinical framework, possible autoimmune encephalitis requires a subacute neuropsychiatric syndrome accompanied by at least one supportive neurologic, cerebrospinal fluid (CSF), seizure-related, or magnetic resonance imaging feature and reasonable exclusion of alternative causes [3]. Classification as autoantibody-negative but probable autoimmune encephalitis requires additional objective support and exclusion of well-characterized neuronal antibodies [3]. Antibody negativity therefore does not exclude an immune-mediated disorder, but neither does it independently establish one. Diagnosis depends on the clinical syndrome, neurologic examination, magnetic resonance imaging, CSF analysis, electroencephalography, and systematic consideration of competing diagnoses [2,3].

Catatonia is a neuropsychiatric syndrome characterized by disturbances in motor activity, behavior, speech, affect, and autonomic function. Although historically associated primarily with schizophrenia, it is now recognized in mood disorders, neurologic disease, autoimmune encephalitis, toxic-metabolic conditions, and general medical illness [4-6]. Current consensus guidance and professional resource documents emphasize systematic assessment, evaluation for underlying medical and neurologic causes, and prompt treatment with benzodiazepines or electroconvulsive therapy when clinically indicated [5,6]. The distinction between antibody negativity and diagnostic confirmation is clinically important because autoimmune encephalitis may be overdiagnosed when nonspecific psychiatric symptoms or treatment responses are interpreted without sufficient objective neurologic support [7]. We describe the longitudinal course of a young woman with a historical clinical diagnosis of possible seronegative autoimmune encephalitis, bipolar I disorder with psychotic features, electroencephalographically documented focal epilepsy, and episodes clinically concerning for recurrent catatonia. The case is notable for the coexistence of several plausible neurologic and psychiatric explanations across multiple hospitalizations, incomplete retrospective documentation of the original autoimmune diagnosis, and evolving diagnostic confidence as later investigations and treatment responses accumulated. It illustrates the value of treating clinically actionable syndromes while repeatedly reassessing autoimmune, seizure-related, medication-related, and primary psychiatric contributors.

## Case presentation

A woman in her early 20s developed an acute neuropsychiatric illness in August 2021 characterized by altered mental status, hallucinations, progressive loss of motor coordination, impaired speech and swallowing, abnormal movements, and catatonia. A mature cystic ovarian teratoma was identified and resected in August 2021. The contemporaneous records described an extensive diagnostic evaluation that was largely unrevealing apart from the teratoma and neuroimaging and metabolic findings considered potentially supportive of an autoimmune process. The treating teams documented that neuronal antibody studies were negative and referred to the illness as possible seronegative, cell-mediated autoimmune encephalitis. Complete original CSF values, including cell count, protein, glucose, oligoclonal bands, IgG index, and the complete serum and CSF antibody panels, were not available in the records reviewed for this report. The records instead summarized the CSF findings as suggestive of a cell-mediated inflammatory process and described the antibody studies as negative. Consequently, the original diagnosis remained clinical rather than serologically confirmed.

During the index illness, the patient also demonstrated severe encephalopathy. This presentation was also marked by prolonged periods of markedly impaired responsiveness, loss of motor function, dysphagia, speech impairment, and autonomic abnormalities. Intermittent sinus tachycardia, at times reaching 130-150 beats per minute, and severe constipation were repeatedly documented and were considered potentially consistent with dysautonomia, although deconditioning, medication effects, anxiety, and pain remained alternative explanations. The patient developed malignant catatonia with profound psychomotor impairment. In September 2021,a formal Bush-Francis Catatonia Rating Scale [5,6] assessment documented a screening score of 17, a severity score of 26, and 12 positive items. Neuroimaging during the 2021 hospitalization was not entirely normal. A brain magnetic resonance imaging study during the 2021 hospitalization was described as showing possible subtle diffuse cortical diffusion restriction with subtle cortical fluid-attenuated inversion recovery hyperintensity, although the radiologist noted that the findings could be artifactual or related to electroconvulsive therapy, prolonged seizure activity, metabolic disturbance, or hypoxic-ischemic injury. Fluorodeoxyglucose positron emission tomography reportedly demonstrated symmetric hypometabolism involving the motor strips, high parietal and occipital regions, and cerebellar hemispheres. Treating neurologists considered the positron emission tomography findings supportive of autoimmune encephalitis, although the findings were not specific. Prolonged video electroencephalography performed early during the 2021 hospitalization did not demonstrate seizures.

Treatment during the index course included teratoma resection and high-dose corticosteroids, followed by a prolonged taper, plasma exchange, intravenous immunoglobulin, and rituximab. Four weekly rituximab infusions were administered over approximately three weeks during the 2021 hospitalization.Prednisone treatment was documented from August 2021 with a gradual taper continuing through November. Because malignant catatonia persisted despite the medical and immunologic treatment course, she underwent 25 electroconvulsive therapy treatments, with the final documented treatment performed later during the 2021 hospitalization. The records described progressively longer periods of lucidity and substantial improvement in catatonia, although improvement could not be attributed to a single intervention because electroconvulsive therapy, olanzapine, prior immunotherapy, corticosteroid treatment, rehabilitation, and the natural course of illness occurred concurrently. Because the original diagnosis was made across multiple institutions and some contemporaneous diagnostic data were unavailable for independent review, the available evidence was compared with the 2016 Graus clinical criteria for possible autoimmune encephalitis and autoantibody-negative but probable autoimmune encephalitis, as summarized in Table 1 [3].

**Table 1:** Application of autoimmune encephalitis diagnostic criteria across the longitudinal course CSF, cerebrospinal fluid; ECT, electroconvulsive therapy; EEG, electroencephalography; FLAIR, fluid-attenuated inversion recovery; MRA, magnetic resonance angiography; MRI, magnetic resonance imaging; MRV, magnetic resonance venography Note: Under the Graus framework, possible autoimmune encephalitis requires rapid progression of altered mental status, memory deficits, or psychiatric symptoms; at least one supportive clinical, CSF, seizure, or MRI feature; and reasonable exclusion of alternative causes. Autoantibody-negative but probable autoimmune encephalitis requires additional objective MRI, CSF, or pathological support and substantiated absence of well-characterized antibodies in serum and CSF [3]. The classifications shown reflect only the documentation available for retrospective review and do not replace the contemporaneous treating teams' clinical judgment.

Diagnostic element	2021 index illness	2023 reassessment	2025 reassessment
Rapid progression within three months of altered mental status, memory impairment, or psychiatric symptoms	Present. Acute onset in August 2021 with hallucinations and altered mental status progressing to impaired coordination, speech, swallowing, responsiveness, and catatonia.	Recurrent mania, psychosis, behavioral dysregulation, and visual symptoms were present, but represented recurrence approximately two years after the index illness rather than a new index encephalitic syndrome.	Recurrent psychosis, bizarre behavior, reduced oral intake, and impaired engagement were present approximately four years after the index illness.
New focal central nervous system findings	Possible. Records described loss of motor coordination, dysphagia, speech impairment, abnormal movements, and severe psychomotor dysfunction, although complete contemporaneous neurologic examinations were unavailable.	No new persistent focal neurologic deficit was established during neurologic reassessment.	No new persistent focal neurologic deficit was established.
Seizures not explained by a preexisting seizure disorder	No seizures were captured ona video EEG performed early during the 2021 hospitalization. Bilateral independent frontotemporal focal epilepsy was subsequently documented in February 2022.	No clinical or electroencephalographic evidence of breakthrough seizures was established during the reassessment.	Later EEGs did not demonstrate epileptiform activity, although episodic seizure activity could not be completely excluded by negative studies.
MRI findings potentially supportive of encephalitis	Equivocal. Brain MRI during the 2021 hospitalization was reported as showing possible subtle diffuse cortical diffusion restriction and cortical FLAIR hyperintensity. The report also identified artifact, ECT-related change, prolonged seizure, metabolic disturbance, and hypoxic-ischemic injury as alternative explanations.	Brain MRI, MRA, and MRV did not demonstrate an acute abnormality supporting active inflammatory recurrence.	Brain imaging did not demonstrate an acute abnormality supporting active inflammatory recurrence.
CSF evidence of inflammation	Complete cell count, protein, glucose, oligoclonal bands, and IgG index were unavailable. Contemporaneous notes summarized the CSF findings as potentially supportive of a cell-mediated inflammatory process, but this could not be independently verified.	Lumbar puncture and CSF autoimmune testing were performed. The complete external results were unavailable in the records reviewed, and no inflammatory recurrence was established by the treating neurologists.	Available records did not document CSF evidence of active inflammatory disease.
Neuronal antibody testing	Records described serum and CSF neuronal antibody studies as negative, but the original reports, tested analytes, and assay details were unavailable. The illness was therefore described clinically as possible seronegative autoimmune encephalitis.	Repeat CSF autoimmune testing was sent externally, but the complete report was unavailable. No serologic confirmation was documented.	No neuronal antibody confirmation of recurrent autoimmune encephalitis was documented.
Alternative causes reasonably excluded	Extensive evaluation was described as largely negative apart from the ovarian teratoma and nonspecific MRI and PET findings. Primary psychiatric illness, cannabis-associated illness, seizure-related phenomena, metabolic causes, and other neurologic disorders remained in the differential. Complete infectious testing was unavailable for independent review.	Neurology considered primary psychiatric illness more likely than breakthrough seizure or autoimmune relapse. Normal imaging and absence of objective inflammatory findings reduced support for active recurrence.	Repeated neurologic evaluation, negative pelvic imaging, absence of objective inflammatory findings, and lack of measurable benefit from IVIG reduced support for active recurrence. Psychiatric illness, catatonia, epilepsy, and medication effects remained competing explanations.
Classification supported by available documentation	The clinical presentation was compatible with possible autoimmune encephalitis, but complete retrospective confirmation was limited by unavailable primary CSF, antibody, infectious, and neurologic data. Criteria for autoantibody-negative but probable autoimmune encephalitis could not be established from the records reviewed.	The available evidence did not establish active autoimmune encephalitis recurrence.	The available evidence did not establish active autoimmune encephalitis recurrence.

**Figure 1:**
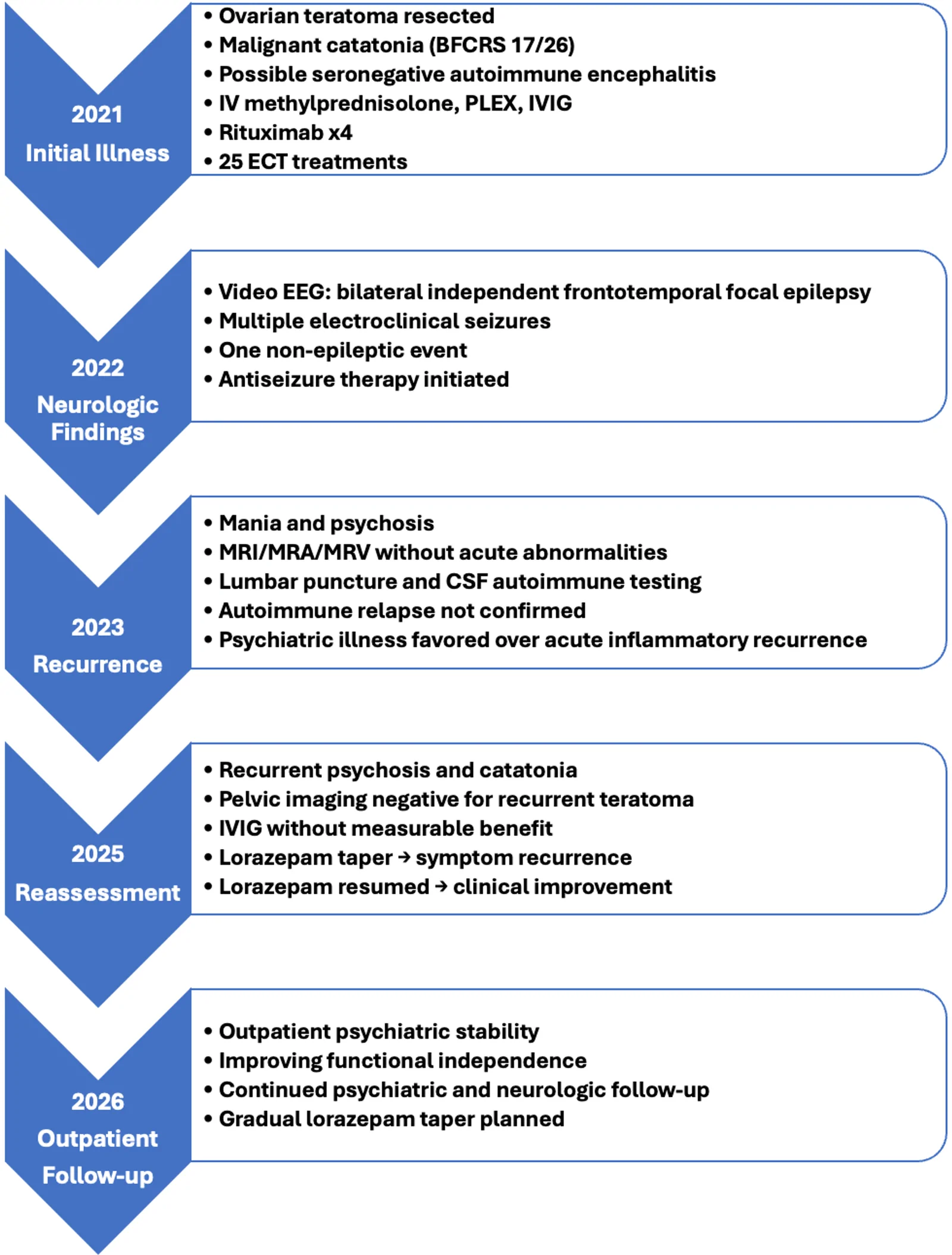
Longitudinal clinical course ECT: electroconvulsive therapy; IVIG: intravenous immunoglobulin; MRI: magnetic resonance imaging Timeline summarizing the patient’s major neurologic and psychiatric events, key objective diagnostic findings, principal therapeutic interventions, and evolution of diagnostic formulation from suspected seronegative autoimmune encephalitis to longitudinal syndrome-based reassessment.

The available documentation therefore explains why autoimmune encephalitis was clinically suspected and treated during the 2021 illness, but it does not permit definitive retrospective classification as autoantibody-negative probable autoimmune encephalitis. In particular, the original CSF values, complete serum and CSF antibody reports, infectious studies, and detailed neurologic examinations were unavailable for independent review. Later reassessments did not reproduce objective findings supporting active inflammatory recurrence. The manuscript consequently uses the term "possible seronegative autoimmune encephalitis" as a historical clinical formulation rather than a confirmed etiologic diagnosis. The patient's longitudinal clinical course is summarized in Figure 1.

To facilitate reconstruction of the longitudinal clinical course, the major hospitalizations, neurologic evaluations, treatments, and treatment responses are summarized chronologically in Table 2.

**Table 2:** Chronological summary of clinical course BFCRS, Bush-Francis Catatonia Rating Scale; CSF, cerebrospinal fluid; CT, computed tomography; ECT, electroconvulsive therapy; EEG, electroencephalography; IVIG, intravenous immunoglobulin; MRA, magnetic resonance angiography; MRI, magnetic resonance imaging; MRV, magnetic resonance venography

Date	Clinical features	Objective investigations	Major interventions	Clinical course/response
Aug 2021	Acute encephalopathy, hallucinations, impaired coordination, dysphagia, speech impairment, abnormal movements	Initial neurologic evaluation; ovarian teratoma identified	Ovarian teratoma resection (August 2021); IV methylprednisolone	Autoimmune encephalitis suspected
Aug-Sep 2021	Progressive neuropsychiatric deterioration	CSF and antibody evaluation (complete reports unavailable)	Plasma exchange; IVIG	Continued severe illness
Sep 2021	Malignant catatonia	BFCRS screening 17; severity 26; 12 positive items	Lorazepam; rituximab ×4	Partial improvement
Sep-Nov 2021	Persistent catatonia	MRI/PET as described	Prednisone taper	Ongoing rehabilitation
Sep-Nov 2021	Persistent malignant catatonia	Clinical monitoring	25 ECT treatments	Substantial recovery
Feb 2022	Episodic behavioral events	Prolonged video EEG: bilateral independent frontotemporal focal epilepsy; multiple electroclinical seizures; one non-epileptic event	Antiseizure therapy initiated	Established seizure disorder
Nov-Dec 2023	Mania, psychosis, visual symptoms	CT head, MRI brain, MRA, and MRV without acute abnormality; neurologic consultation	Lithium, oxcarbazepine, and antipsychotic therapy	Active autoimmune relapse and breakthrough seizure were not established; psychiatric illness favored
Sep 2025	Psychosis, bizarre behavior, reduced oral intake, withdrawal, psychomotor slowing, concern for catatonia	Neurologic reassessment without objective evidence of active inflammatory disease	IVIG for five days; lorazepam initiated	No measurable IVIG benefit; clinically suspected catatonic features improved
Sep 2025	Recurrent psychosis/catatonia	CT abdomen/pelvis and pelvic ultrasound negative for recurrent teratoma	Court-authorized treatment; long-acting antipsychotics	Persistent psychiatric stabilization required
Late 2025	Medication adverse effects	Routine laboratory monitoring	Fluphenazine discontinued for extrapyramidal symptoms; benztropine added; valproate discontinued because of sedation	Improved alertness and resolution of involuntary eye movements
2026 follow-up	Outpatient psychiatric stability	No objective evidence supporting active inflammatory relapse	Continued antipsychotics, antiseizure therapy, scheduled lorazepam	Improving functional independence; gradual lorazepam taper planned

**Figure 2:**
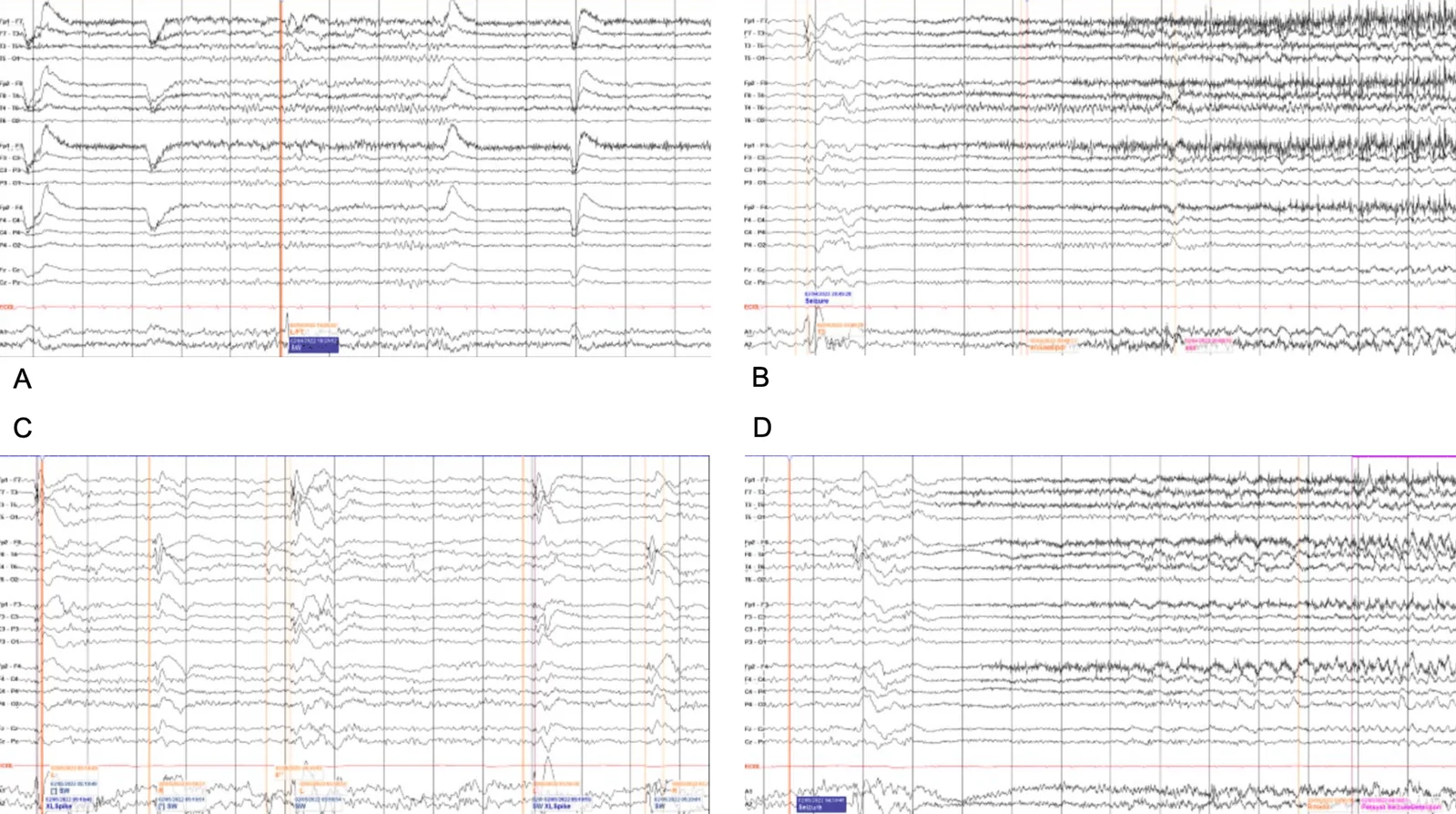
Representative prolonged video electroencephalography demonstrating bilateral independent frontotemporal focal epilepsy (A) Interictal sharp waves during wakefulness. (B) Electroclinical seizure arising from the left hemisphere. (C) Independent interictal epileptiform activity arising from the left and right hemispheres during sleep. (D) Electroclinical seizure arising from the right hemisphere. Collectively, these representative tracings demonstrate bilateral independent epileptiform activity with electroclinical seizures arising from both hemispheres.

In 2022, prolonged video electroencephalography demonstrated bilateral independent frontotemporal focal epilepsy with multiple electroclinical seizures and one non-epileptic event. During one electroclinical seizure, the patient reported a visual hallucination. Representative electroencephalographic findings from the prolonged video EEG are shown in Figure 2.

**Figure 3:**
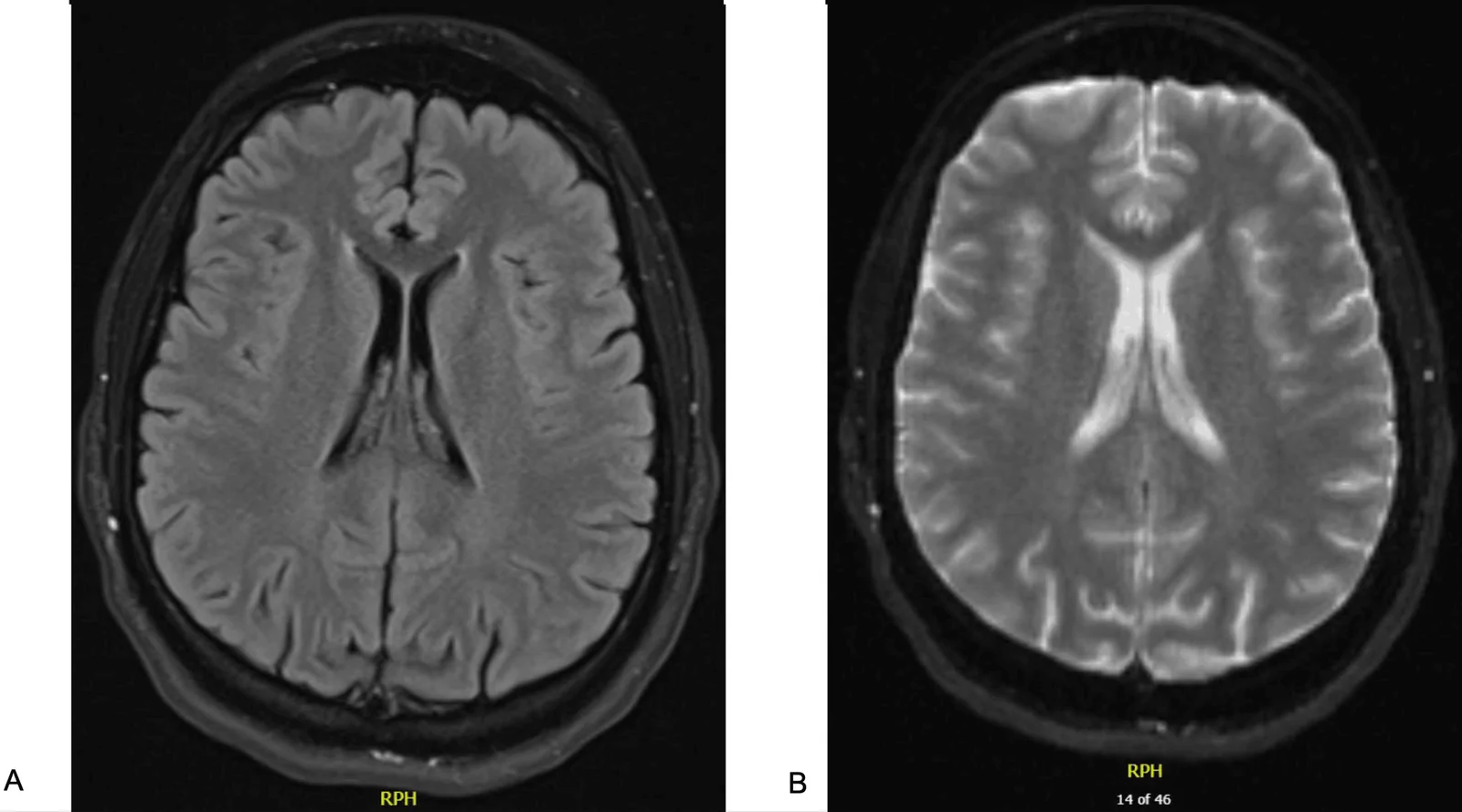
Representative brain magnetic resonance imaging during neurologic reassessment (A) Axial fluid-attenuated inversion recovery (FLAIR) image demonstrating no acute intracranial abnormality. (B) Axial diffusion-weighted imaging (DWI) demonstrating no diffusion restriction. These representative images were obtained during evaluation of recurrent neuropsychiatric symptoms and demonstrated no acute intracranial abnormality or diffusion restriction.

In November 2023, the patient was admitted psychiatrically with mania and psychosis characterized by pressured and rapid speech, flight of ideas, tangential thought processes, affective lability, paranoia, psychomotor agitation, severe thought disorganization, and poor insight. Treatment included lithium, oxcarbazepine, and antipsychotic therapy. During the hospitalization, she developed progressive visual symptoms and was transferred to a medical service during the December 2023 hospitalization for neurologic reassessment. Computed tomography of the brain demonstrated no acute intracranial abnormality, and brain magnetic resonance imaging, magnetic resonance angiography, and magnetic resonance venography did not demonstrate findings supporting active inflammatory recurrence. Neurology considered recurrent autoimmune encephalitis, breakthrough seizure activity, and primary psychiatric illness, but ultimately regarded a psychiatric explanation as more likely than active autoimmune relapse or seizure-related deterioration. She was medically cleared and discharged later during the December 2023 hospitalization, with outpatient psychiatric follow-up. Upon starting an intensive outpatient program, she initially demonstrated partial improvement before developing recurrent altered mental status and psychiatric deterioration characterized by paranoia, severe thought disorganization, guarded behavior, intermittent refusal of oral intake, impaired engagement, and psychomotor slowing. Recurrent autoimmune encephalitis and catatonia remained competing diagnostic considerations, prompting transfer from psychiatry to a medical service for additional neurologic evaluation, including brain magnetic resonance imaging, lumbar puncture, and CSF autoimmune antibody testing submitted to an external laboratory. Representative magnetic resonance imaging obtained during neurologic reassessment is shown in Figure 3.

The major objective diagnostic investigations performed throughout the patient's longitudinal clinical course are summarized in Table 3, and selected laboratory findings relevant to clinical management are summarized in Table 4.

**Table 3:** Objective diagnostic evaluation across the longitudinal clinical course Abbreviations: CT, computed tomography; EEG, electroencephalography; MRA, magnetic resonance angiography; MRI, magnetic resonance imaging; MRV, magnetic resonance venography Electroencephalography findings are summarized from the prolonged video EEG of February 2022 and subsequent follow-up studies.

Investigation	2021-2022 Index Illness	2023 Reassessment	2025 Reassessment
Brain CT	No acute intracranial abnormality	No acute intracranial abnormality	-
Brain MRI	Equivocal possible subtle diffuse cortical diffusion restriction and cortical FLAIR hyperintensity; artifact and several noninflammatory explanations were identified in the report.	No acute intracranial abnormality	No acute intracranial abnormality
MRA Head/Neck	-	No significant abnormality	-
MRV Head	-	No significant abnormality	-
EEG	February 2022 prolonged video EEG demonstrated independent bilateral frontotemporal focal epilepsy with multiple electroclinical seizures and one nonepileptic event	No epileptiform activity	No epileptiform activity
Pelvic imaging	Mature cystic ovarian teratoma identified and resected	-	CT abdomen/pelvis and transvaginal ultrasound negative for recurrent teratoma

**Table 4:** Selected laboratory findings relevant to clinical management Abbreviations: HbA1c, hemoglobin A1c; MHD, monohydroxy derivative; THC, tetrahydrocannabinol; TSH, thyroid-stimulating hormone. *The reference ranges shown are commonly used adult reference intervals and therapeutic ranges; values may vary slightly among laboratories.

Laboratory parameter	Common reference range*	2021	2023	2025
Sodium	135-145 mmol/L	138 mmol/L	131-133 mmol/L	135-139 mmol/L
White blood cell count	4.0-11.0 × 10⁹/L	Within normal limits	Within normal limits	13.2 × 10⁹/L on admission; normalized during hospitalization
Lithium	0.6-1.2 mmol/L (therapeutic)	-	0.80 mmol/L	0.27 → 1.02 → 0.48 mmol/L
Oxcarbazepine metabolite (MHD)	10-35 μg/mL (therapeutic)	-	25-34 μg/mL	-
TSH	0.4-4.0 μIU/mL	-	2.714 μIU/mL	-
HbA1c	4.0-5.6%	-	-	4.8%
Urine toxicology	Negative	THC-positive; otherwise negative	Negative	Negative

Her next major psychiatric hospitalization occurred during a seven-week period in late 2025. She again developed severe psychosis, bizarre behavior, impaired engagement, and markedly reduced oral intake, prompting transfer to a medical service for additional neurologic evaluation. The neurologic workup did not establish recurrent inflammatory disease. A five-day course of intravenous immunoglobulin was administered because autoimmune encephalitis relapse remained under consideration, but no measurable clinical benefit was documented. Lorazepam was initiated at 0.5 mg three times daily because withdrawal, reduced oral intake, psychomotor slowing, behavioral negativism, and impaired engagement raised clinical concern for catatonia. Formal Bush-Francis Catatonia Rating Scale scoring was not documented during this episode. These features subsequently improved, and the treating team described the catatonic syndrome as having resolved. Lorazepam was tapered and discontinued before discharge.

Shortly after discharge, the patient again deteriorated with bizarre behavior, severe thought disorganization, tangential and nonsensical speech, poor oral intake, medication refusal, and inability to sustain coherent conversation, prompting readmission to inpatient psychiatry. Neurology reportedly did not recommend further neurologic investigation during that admission. Computed tomography of the abdomen and pelvis and pelvic ultrasonography demonstrated no evidence of recurrent ovarian teratoma. Persistent psychosis, poor insight, medication refusal, and behavioral dysregulation required civil commitment and court-authorized treatment over objection. Antipsychotic therapy was intensified and ultimately transitioned to long-acting injectable formulations to improve symptom control and adherence. Lorazepam was reintroduced because of renewed clinical concern for catatonia and was titrated to a scheduled dose of up to 2 mg four times daily. Formal Bush-Francis Catatonia Rating Scale scores were not available during these later episodes. Attempts to taper or discontinue lorazepam were followed by recurrence of withdrawal, reduced oral intake, severe disorganization, impaired engagement, and other features considered potentially catatonic. Re-initiation or as-needed administration was temporally associated with improvement, although concurrent antipsychotic changes, environmental structure, nutritional support, and the natural course of illness limited causal interpretation.

A subsequent hospitalization occurred after the patient developed aggressive behavior, inability to be redirected, refusal to engage, loud and profane speech, and reported suicidal ideation while under outpatient commitment. Schizophrenia with catatonia was documented during that admission, although her broader longitudinal record continued to include bipolar I disorder with psychotic features, suspected seronegative autoimmune encephalitis, focal epilepsy, and recurrent catatonia as competing or overlapping diagnostic formulations. Before discharge, she stabilized on dual antipsychotic therapy, mood-stabilizing treatment, and scheduled lorazepam.

Following discharge, she remained under outpatient commitment with ongoing psychiatric and neurologic follow-up. Long-acting injectable fluphenazine was later discontinued because of involuntary eye movements concerning extrapyramidal symptoms, which improved after treatment with as-needed benztropine and discontinuation of fluphenazine. Valproate, prescribed for seizure management in addition to scheduled oxcarbazepine, was discontinued by neurology because of excessive daytime sedation, with subsequent improvement in alertness.

At the most recent outpatient follow-up, the patient denied acute psychiatric symptoms, attended appointments independently, and demonstrated increasing functional independence. She remained engaged in psychiatric and neurologic care while continuing antipsychotic, antiseizure, and scheduled lorazepam therapy. Gradual lorazepam dose reduction was being considered because of daytime sedation, although continued treatment was favored because of her history of malignant catatonia and the observed temporal relationship between lorazepam administration and recurrent clinical improvement.

## Discussion

This case illustrates the difficulty of managing recurrent neuropsychiatric illness when autoimmune, seizure-related, medication-related, and primary psychiatric explanations remain simultaneously plausible. The patient's history of suspected seronegative autoimmune encephalitis, electroencephalographically documented focal seizures, visual and optic nerve abnormalities, severe psychiatric symptoms, and prior malignant catatonia appropriately prompted repeated neurologic reassessment [1-3]. Her ovarian teratoma also remained clinically relevant because ovarian teratomas are associated with N-methyl-D-aspartate receptor (NMDAR)-antibody encephalitis and may contain NMDAR-specific germinal-center activity [8]. These historical features justified evaluation for teratoma recurrence and contributed to the decision to administer intravenous immunoglobulin during a later hospitalization.

Autoimmune encephalitis may initially present with prominent psychiatric symptoms, including psychosis, mood disturbance, agitation, disorganization, and catatonia [1,2]. Diagnostic frameworks, therefore, caution against excluding the disorder solely because neuronal autoantibodies are absent [2,3]. Seronegativity, however, does not itself establish an autoimmune etiology. Diagnosis requires a compatible clinical syndrome supported by neurologic examination, neuroimaging, electroencephalography, cerebrospinal fluid findings, or other objective evidence, together with adequate consideration of competing diagnoses [3]. This distinction is important because autoimmune encephalitis may be overdiagnosed, particularly when nonspecific psychiatric symptoms are interpreted without sufficient objective neurologic support. Studies of autoimmune encephalitis misdiagnosis have described patients whose eventual diagnoses included alternative neurologic, functional, and primary psychiatric disorders [7].

In this patient, the degree of diagnostic certainty evolved over time. The original illness was clinically compelling because encephalopathy, abnormal movements, severe catatonia, an ovarian teratoma, and subsequent focal epilepsy occurred within the same longitudinal course. Later episodes, however, were not accompanied by consistent evidence of renewed inflammatory disease. Subsequent magnetic resonance imaging, vascular imaging, cerebrospinal fluid evaluation, neurologic consultation, and pelvic imaging did not establish active autoimmune recurrence or recurrent teratoma. Electroencephalograms obtained after the period of documented focal epilepsy did not demonstrate recurrent epileptiform activity, and intravenous immunoglobulin administered during a later episode produced no measurable clinical benefit. These findings did not invalidate the historical diagnosis, but they reduced support for attributing every subsequent psychiatric deterioration to recurrent encephalitis. The appropriate response was therefore neither to dismiss the original autoimmune formulation nor to treat it as a fixed explanation for all later symptoms. Instead, diagnostic confidence required repeated revision as examination findings, investigations, treatment responses, and specialist assessments accumulated.

Catatonia became an increasingly important syndromic formulation because similar constellations of withdrawal, markedly reduced oral intake, psychomotor slowing, behavioral negativism, impaired engagement, and severe functional decline recurred across multiple hospitalizations. These features were compatible with catatonia but were not specific and overlapped with severe psychosis, mood disturbance, medication effects, delirium, postictal states, and behavioral refusal. Standardized Bush-Francis Catatonia Rating Scale scores [5,6] were documented during the 2021 malignant catatonia episode but were unavailable during later hospitalizations, limiting certainty that each later deterioration represented catatonia. Lorazepam-associated improvement was observational and may also have reflected effects on anxiety, agitation, insomnia, manic activation, or nonspecific behavioral dysregulation. Catatonia may occur in mood disorders, psychotic disorders, neurologic disease, and autoimmune encephalitis, including anti-NMDAR encephalitis [4-6,9]. Recognizing catatonia, therefore, did not resolve the underlying etiology. It did, however, identify a clinically actionable and potentially reversible syndrome.

The temporal relationship between lorazepam treatment and symptom improvement was particularly important. During the medical admission, the patient reportedly improved after lorazepam was initiated, deteriorated after it was tapered or discontinued, and improved again when treatment was resumed. Her earlier malignant catatonia had also responded to electroconvulsive therapy. Although the temporal association between lorazepam administration and improvement was clinically important, benzodiazepine responsiveness was interpreted as supportive rather than diagnostic of catatonia. Lorazepam may also reduce anxiety, agitation, insomnia, manic activation, and nonspecific behavioral dysregulation. Improvement may likewise have reflected concurrent changes in antipsychotic therapy, supportive inpatient care, nutritional rehabilitation, environmental structure, or the natural evolution of illness. Consequently, treatment response was interpreted within the broader longitudinal clinical context rather than as evidence establishing the underlying etiology.

The British Association for Psychopharmacology consensus guideline recommends benzodiazepines, particularly lorazepam, as first-line treatment for catatonia and supports electroconvulsive therapy for malignant, severe, or benzodiazepine-resistant presentations [5]. The American Psychiatric Association Resource Document similarly reviews benzodiazepines and electroconvulsive therapy as principal treatments while emphasizing individualized assessment and management [6]. The British Association for Psychopharmacology guideline also advises caution when tapering benzodiazepines in recurrent or persistent cases because relapse may follow dose reduction or discontinuation [5]. Recent evidence similarly identifies recurrent and relapsing catatonia as a clinically significant problem across psychotic and affective disorders [10]. The patient's repeated deterioration during lorazepam reduction is consistent with these recognized patterns, although causality cannot be established from a single observational case.

The case also demonstrates the importance of distinguishing catatonic deterioration from persistent psychosis, mania, seizure-related behavioral change, and medication adverse effects. Pressured speech, flight of ideas, paranoia, affective lability, agitation, and severe disorganization supported an ongoing mood or psychotic process. In contrast, withdrawal, reduced oral intake, psychomotor slowing, negativism, and diminished engagement raised concern for catatonia. These syndromes overlapped and required simultaneous treatment rather than a single explanatory diagnosis. The patient's documented bilateral independent frontotemporal focal epilepsy added further diagnostic complexity. Prolonged video electroencephalography performed in February 2022 demonstrated multiple electroclinical seizures arising independently from both hemispheres, confirming an objective seizure disorder. Psychiatric manifestations associated with focal epilepsy may include ictal behavioral phenomena, postictal psychosis, chronic interictal psychosis, affective instability, and behavioral disturbance, particularly in patients with frontal or temporal lobe epilepsy [11]. Although these possibilities remained part of the longitudinal differential diagnosis, several observations reduced support for seizure-related psychiatric syndromes as the primary explanation for the later hospitalizations. Subsequent electroencephalography did not demonstrate recurrent epileptiform activity, although the absence of captured epileptiform activity did not establish that all later behavioral episodes were unrelated to epilepsy. The prolonged psychiatric episodes also lacked a consistent temporal relationship with documented seizures, and symptom recurrence appeared more closely associated with changes in lorazepam therapy than with changes in antiseizure treatment. Nevertheless, seizure-related behavioral syndromes, medication effects, and autoimmune encephalitis remained plausible competing contributors throughout the patient's course, reinforcing the need for repeated neurologic reassessment rather than attribution of all psychiatric deterioration to a single diagnosis.

Antipsychotic treatment remained necessary because of persistent psychosis, disorganization, and medication refusal, but it required careful monitoring. Dopamine-blocking medications may worsen catatonia or complicate differentiation from neuroleptic malignant syndrome in susceptible patients [5,6]. Medication adverse effects further obscured the clinical picture. Long-acting fluphenazine was associated with involuntary eye movements concerning extrapyramidal symptoms, prompting discontinuation and treatment with benztropine. Valproate contributed to substantial daytime sedation that improved after the medication was stopped. Such effects may mimic worsening encephalopathy, negative symptoms, psychomotor slowing, or catatonia. Repeated medication reconciliation and reassessment of cumulative treatment burden were therefore essential components of longitudinal care.

**Figure 4:**
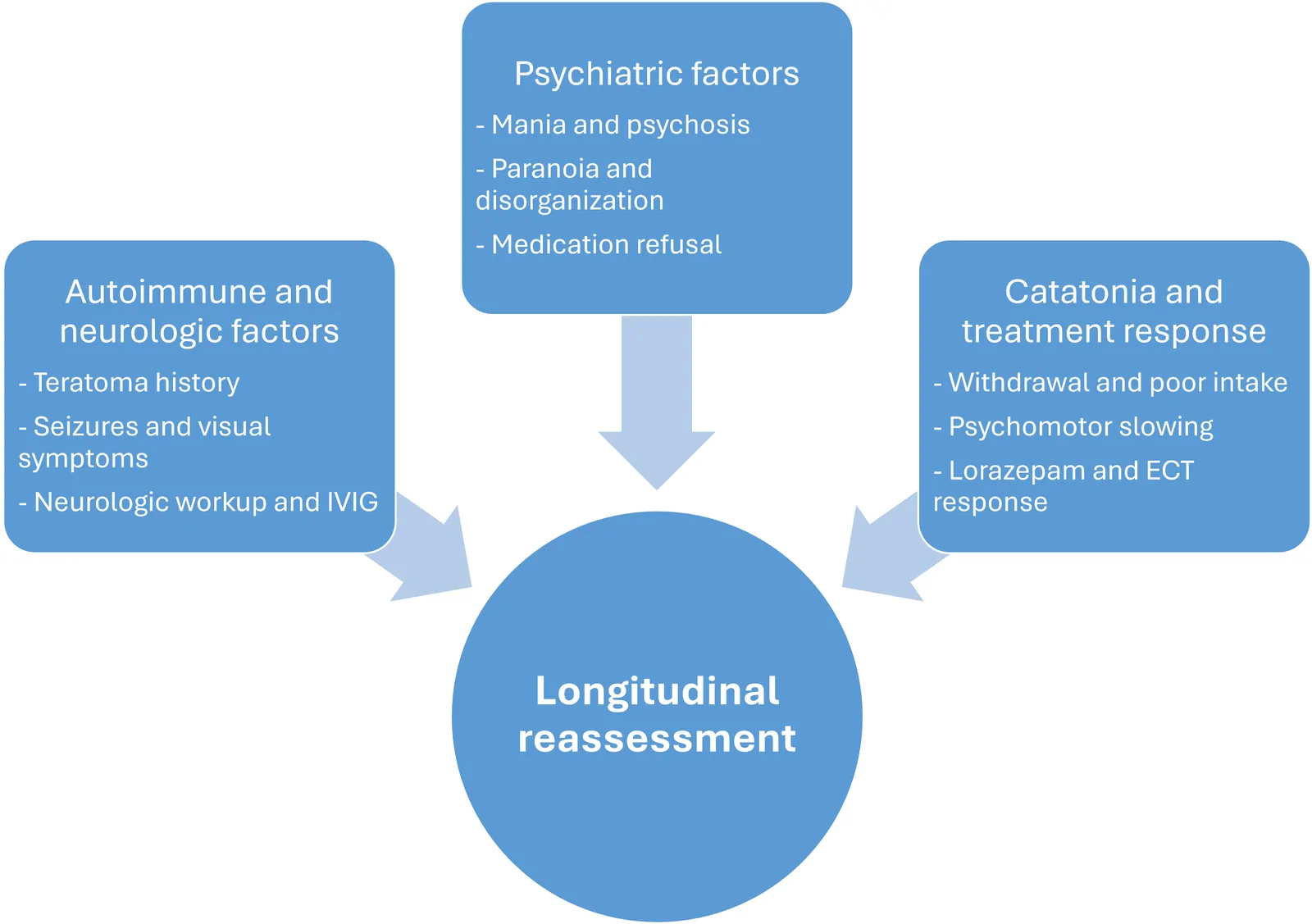
Longitudinal factors guiding diagnostic reassessment Recognition and treatment of catatonia continued while autoimmune, neurologic, and primary psychiatric contributors remained under evaluation.

More broadly, this case demonstrates the risks of diagnostic anchoring in both directions. Exclusive attribution of recurrent symptoms to autoimmune encephalitis could lead to repeated immunotherapy without adequate treatment of catatonia, psychosis, or medication-related complications. Conversely, exclusive attribution to a primary psychiatric disorder could cause clinicians to overlook seizures, inflammatory disease, tumor recurrence, or other neurologic contributors. A syndrome-based approach allowed clinicians to treat the immediately actionable manifestations, particularly catatonia and psychosis, while continuing to reassess competing etiologies. Figure 4 summarizes the longitudinal clinical features, objective neurologic findings, treatment-response patterns, and competing diagnostic considerations that informed diagnostic reassessment throughout the patient’s clinical course.

Several limitations should be acknowledged. The original diagnosis of autoimmune encephalitis remained clinically suspected rather than serologically confirmed, and the complete external neuronal antibody data were not available for independent review. Although the historical presentation was compatible with seronegative autoimmune encephalitis, later hospitalizations did not yield definitive evidence of active inflammatory relapse. The relationship between lorazepam tapering, symptom recurrence, and improvement after re-initiation was observational and may have been influenced by simultaneous changes in antipsychotic, mood-stabilizing, antiseizure, environmental, or supportive treatment. Standardized Bush-Francis Catatonia Rating Scale scores [5,6] were documented during the index episode but were not consistently repeated during later admissions, limiting objective comparison of catatonia severity across time. In addition, the longitudinal record was assembled across multiple institutions, and some original diagnostic studies were available only in summarized reports or as incorporated images, rather than through direct access to the originating systems.

Despite these limitations, the longitudinal course provides a useful clinical lesson. Historical evidence of autoimmune encephalitis should remain relevant when new neuropsychiatric symptoms emerge, but it should not preclude reconsideration when subsequent objective findings and treatment responses point elsewhere. In this case, evolving diagnostic certainty and a syndrome-based treatment strategy allowed clinicians to preserve appropriate concern for neurologic disease while addressing recurrent catatonia, psychosis, seizures, and medication-related complications on their own clinical merits.

## Conclusions

This case demonstrates that episodes clinically concerning for recurrent catatonia may remain actionable even when their underlying etiology cannot be definitively established. The patient's history of ovarian teratoma, clinically suspected seronegative autoimmune encephalitis, electroencephalographically documented focal epilepsy, and severe neuropsychiatric illness appropriately warranted continued neurologic reassessment. Later investigations did not consistently demonstrate active inflammatory relapse. Repeated temporal associations between lorazepam administration and improvement, deterioration during tapering, and prior response of malignant catatonia to electroconvulsive therapy supported continued catatonia-directed management, although benzodiazepine responsiveness was not diagnostic, and later episodes lacked serial standardized catatonia ratings.

Complex neuropsychiatric presentations rarely conform to a single diagnostic framework. Longitudinal integration of psychiatric phenomenology, neurologic findings, objective diagnostic studies, medication effects, collateral information, and treatment response may yield greater diagnostic accuracy than conclusions drawn from a single hospitalization. Historical evidence of autoimmune encephalitis should remain an important consideration, but should not preclude revising the diagnostic formulation as new evidence emerges. Recognition and treatment of clinically actionable syndromes such as catatonia should proceed while competing autoimmune, neurologic, and primary psychiatric etiologies continue to be evaluated. This case highlights the value of longitudinal reassessment in avoiding diagnostic anchoring and guiding management of recurrent neuropsychiatric illness.
